# Mutations in the Ferric Uptake Regulator Gene (*fur*) Suppress the Bacitracin Sensitivity of a *Helicobacter pylori fapH* Deletion Mutant

**DOI:** 10.3390/microorganisms13092103

**Published:** 2025-09-09

**Authors:** Kyle Rosinke, Timothy R. Hoover

**Affiliations:** Department of Microbiology, University of Georgia, Athens, GA 30602, USA; krosinke@uga.edu

**Keywords:** *Helicobacter pylori*, flagellum, flagellar motor, outer membrane, motility

## Abstract

*Helicobacter pylori* uses a cluster of polar flagella for motility. *H. pylori* FapH forms a ring-like flagellar motor accessory associated with the outer membrane. A *H. pylori* Δ*fapH* mutant displays a motility-dependent sensitivity to bacitracin, an antibiotic that is normally excluded by the outer membrane, which suggests that FapH helps to maintain the integrity of the outer membrane during flagellar rotation. We report here that deletion of the ferric uptake regulator (*fur*) gene suppressed the bacitracin sensitivity of the *H. pylori* Δ*fapH* mutant. Depleting intracellular iron in the *H. pylori* Δ*fapH* mutant with the iron chelator 2,2′-dipyridyl similarly suppressed the bacitracin sensitivity of the strain. We postulate the altered expression of Fur-regulated genes as a result of deleting *fur* or that iron deprivation suppressed the bacitracin sensitivity of the Δ*fapH* mutant. We also isolated two bacitracin-resistant Δ*fapH* strains that had a nonsense mutation in *lpxF*, which encodes a lipid A 4′-phosphatase. Loss of LpxF alters the structure of the lipid A backbone in lipopolysaccharide that stabilizes the outer membrane, which we hypothesize compensated for the loss of FapH by minimizing damage to the membrane resulting from flagellar rotation.

## 1. Introduction

*Helicobacter pylori* is a Gram-negative bacterium that is estimated to infect the gastric mucosae of about half of the human population worldwide [1]. While most individuals infected with *H. pylori* are asymptomatic, *H. pylori* infections can result in chronic gastritis and peptic ulcer disease, and they are a significant risk factor for the development of gastric cancer [2,3,4]. A recent review by Malfertheiner and co-workers provides a broad overview of the history, epidemiology, pathophysiology, diagnosis, and management of *H. pylori* infection [5].

*H. pylori* uses a cluster of polar flagella for swimming motility, and studies in animal models indicate that flagellum-mediated motility is required for host colonization by the bacterium [6,7]. One of the characteristic features of the *H. pylori* flagellum is the membranous sheath that surrounds the flagellar filament and is contiguous with the outer membrane (OM) [8].

The bacterial flagellum is a nanomachine that consists of three main sections—the basal body, a hook, and a filament. The basal body houses a rotary motor consisting of torque-generating stator units, a rotor, a driveshaft, and a bushing/bearing complex. In *Escherichia coli* and other bacteria, the stator units are comprised by the membrane proteins MotA and MotB, which form a transmembrane ion channel that allows the influx of protons to drive the rotation of the MotA pentameric ring [9,10,11,12]. In *Vibrio alginolyticus* and other marine bacteria, the stator units are formed by the MotA/MotB homologs PomA and PomB, which utilize a sodium ion gradient to generate torque [13,14]. The rotor consists of a cytoplasmic C-ring and a transmembrane MS-ring formed by FliF [15,16]. The C-ring is formed by FliG, FliM, and FliN in the *E. coli* motor, while in *H. pylori*, the C-ring is comprised of these three proteins plus FliY, which is homologous with FliN [17,18]. The rod is mounted on the MS-ring and acts as a driveshaft to transmit torque from the rotor to the filament via the hook. The LP-ring complex serves as a bushing that aligns and balances the rod as it rotates [16,19,20]. In *E. coli* and *Salmonella enterica*, the P-ring portion of the LP-ring complex is associated with the peptidoglycan layer, while the L-ring forms a pore in the OM through which the rod passes [16,19]. The hook is a flexible rod-like structure that allows for the transmission of torque to the filament in a non-axial orientation relative to the rod [21,22]. The filament is a rigid helical structure that propels the cell forward as it rotates [23].

In addition to the core structures found in the archetypal *E. coli* and *S. enterica* flagellar motors, the *H. pylori* motor possesses additional motor accessories [24,25,26,27,28,29,30]. Proposed roles for the *H. pylori* motor accessories include the recruitment and retention of stator units, stabilization of stator–rotor interactions, and protection of the cell envelope from the high torque generated by the motor [24,27,31,32]. Depending on the species, the flagellar motor is capable of rotating from hundreds to more than one thousand revolutions per second [33]. As the flagellum rotates, the cell body of the bacterium rotates in the opposition direction [33]. The rotation of the flagellum and counterrotation of the cell body are potential stressors on the OM, and this stress may be exacerbated in *H. pylori* and other bacteria that have sheathed flagella. Consistent with this hypothesis, rotation of the sheathed flagellum is a significant generator of OM vesicles (OMVs) in *Vibrio* species [34,35]. In addition to having a role in OMV biogenesis, rotation of the sheathed flagellum may disrupt the lipid asymmetry of the OM. The outer leaflet of the OM is composed of lipopolysaccharide (LPS), while the inner leaflet is formed of phospholipids. The OM is an effective barrier to many toxic compounds, and disrupting the lipid asymmetry of the OM increases the permeability of the OM to such compounds [36]. Thus, flagellar rotation and cell body counterrotation may compromise the barrier function of the OM by facilitating the flipping of phospholipids into the OM’s outer leaflet.

Consistent with the hypothesis that some of the *H. pylori* motor accessories protect the OM from flagellum-mediated damage, a ring structure in the *H. pylori* motor that is located near the OM appears to protect the barrier function of the OM during flagellar rotation [28]. A lipoprotein designated as FapH (flagellum-associated protein in *Helicobacter pylori*) is proposed to form the subunits of the ring. Deletion of *fapH* in *H. pylori* B128 results in increased sensitivity to bacitracin [28], an antibiotic that inhibits peptidoglycan synthesis and is normally excluded by the OM. A *H. pylori* Δ*fapH* mutant that has paralyzed flagella due to a mutation in *pflA* displays wild-type resistance to bacitracin, indicating that the bacitracin sensitivity of the Δ*fapH* mutant is dependent on flagellar rotation [28]. These findings suggest that in the absence of the FapH-ring, the barrier function of the OM is compromised during flagellar rotation, presumably resulting from the intrusion of phospholipids into the outer leaflet of the OM and/or sheath.

To examine further the molecular basis for how loss of FapH renders *H. pylori* sensitive to bacitracin, we characterized twelve independent isolates of a *H. pylori* B128 Δ*fapH* mutant that had increased resistance to bacitracin. *H. pylori* has a high mutation rate and secondary mutations often arise during the construction of deletion mutants. This was the case for the *H. pylori* Δ*fapH* strain from which the bacitracin-resistant isolates were derived, as it has a mutant *pflA* (paralyzed flagella protein A) allele (which was designated as pflA* in a previous report) that encodes a PflA variant in which a twelve-amino acid sequence is altered [28]. The *H. pylori* ΔfapH pflA* mutant displays robust motility in soft agar medium, and the predicted tertiary structure of the PflA variant encoded by the pflA* allele is indistinguishable from that of the native PflA [28]. All of the bacitracin-resistant ΔfapH pflA* isolates had a mutation in the ferric uptake regulator (*fur*) gene. *H. pylori* Fur is a global regulator that affects expression of genes involved in iron homeostasis as well as several other genes that do not have roles in iron metabolism [37,38,39]. Deleting *fur* in the *H. pylori* ΔfapH pflA* mutant suppressed the bacitracin sensitivity of the strain, as did depletion of intracellular iron by the addition of the ferrous iron chelator 2,2′-dipyridyl to the growth medium.

In constructing a *H. pylori* B128 Δ*fapH* mutant that lacked secondary mutations in any known flagellar genes, we previously generated several Δ*fapH* isolates [28]. Two of these Δ*fapH* isolates were significantly more resistant to bacitracin compared to the other Δ*fapH* isolates, and both of the bacitracin-resistant Δ*fapH* isolates had the same nonsense mutation in *lpxF*. LpxF is a phosphatase that removes the 4′-phosphate from the lipid A backbone during the biosynthesis of LPS [40]. Failure to remove the 4′-phosphate from lipid A interferes with the subsequent removal of a phosphatidic acid group from the lipid A backbone by LpxR [40,41]. We hypothesize that the altered structure of the lipid A backbone in the absence of LpxF strengthened the OM and mitigated the stress placed on the OM resulting from flagellar rotation in the Δ*fapH* mutants.

## 2. Materials and Methods

### 2.1. Bacterial Strains and Culture Conditions

For routine cloning procedures, *E. coli* Turbo cells (New England Biolabs, Ipswich, MA, USA) were grown on LB medium supplemented with ampicillin (100 μg/mL) (Cellgro, Herndon, VA, USA) or kanamycin (30 μg/mL) (Gold Biotechnology, St. Louis, MO, USA) as needed. The *H. pylori* strains and plasmids used in the study are listed in Table S1. Liquid cultures of *H. pylori* were grown at 37 °C with shaking in either brain–heart infusion medium (Becton, Dickinson and Company, Sparks, MD, USA) or Mueller–Hinton broth (Hardy Diagnostics, Santa Maria, CA, USA), both supplemented with 5% heat-inactivated horse serum (Gibco; Thermo Fisher Scientific, Waltham, MA, USA). *H. pylori* cultures in liquid medium were grown in sealed glass serum vials that contained an atmosphere consisting of 5% CO_2_, 10% H_2_, 10% O_2_, and 75% N_2_. For growth on solid medium, *H. pylori* strains were grown on tryptic soy agar supplemented with 5% heat-inactivated horse serum (TSA-HS) at 37 °C under an atmosphere consisting of 10% CO_2_, 8% O_2_, and 82% N_2_. Growth media for *H. pylori* were supplemented with kanamycin (30 μg/mL), bacitracin (200 μg/mL), polymyxin B (3, 10, 40 μg/mL), 2-2′ dipyridyl (25, 50, 75, 100 μM), or 5% sucrose (*w*/*v*) as needed. Bacitracin, polymyxin B, 2-2’-dipyridyl, and sucrose were purchased from Sigma-Aldrich, St. Louis, MO, USA.

### 2.2. PCR Methods

PrimeSTAR DNA polymerase (Takara Bio, San Jose, CA, USA) or Phusion DNA polymerase (New England Biolabs, Ipswich, MA, USA) were used to amplify DNA from *H. pylori* B128 genomic DNA (gDNA), which was purified using the Wizard genomic DNA purification kit (Promega, Madison, WI, USA). The resulting amplicons were incubated with *Taq* polymerase (Promega, Madison, WI, USA) at 72 °C for 10 min to add A-overhangs at the 3′-ends and then cloned into the pGEM-T Easy vector (Promega, Madison, WI, USA). PCR primers were obtained from Integrated DNA Technologies, Coralville, IA, USA.

### 2.3. Construction of H. pylori B128 Δfur Mutants

Flanking sequences of *fur* were amplified from *H. pylori* B128 gDNA using the primer pair *fur*_US_F (5′-TTTCAGTCAAACAAATCGGCTA-3′) and *fur*_US-R (5′-GAATTCGATTATCCTCGAGGCTGATATCTTCCTTATCCGTAAAA-3′) for the upstream region and the primer pair *fur*_DS-R (5′-TGTAGAGTTGCCTGGAATTTATC-3′) and *fur*_DS_F (5′-GATAATCGAATTCGCTAGCAAAGAAGAAGCTTAGATAGGGC-3′) for the downstream region. The 5′-ends of *fur*_US-R and *fur*_DS_F are complementary and introduced XhoI and NheI restriction sites. The PCR products were joined by overlapping PCR, and following the addition of A-overhangs to its 3′-ends the resulting amplicon was cloned into pGEM-T Easy to generate plasmid pKR158. A kan^R^*-sacB* cassette in plasmid pKR3 [28] was excised and cloned into the NheI and XhoI sites in plasmid pKR158 to generate the suicide vector pKR159. Plasmid pKR159 was introduced by natural transformation into wild-type *H. pylori* B128 and the *H. pylori* B128 ΔfapH pflA* mutant by natural transformation. Transformants in which *fur* had been replaced with the kan^R^-*sacB* cassette were isolated by selecting for kanamycin-resistant colonies. Replacement of the chromosomal copy of *fur* with the kan^R^-*sacB* cassette was confirmed by PCR using the primer pair *fur*_US-F and *fur*_DS_R. The suicide vector pKR158 was introduced into isolates of wild-type *H. pylori* B128 and the ΔfapH pflA* mutant, in which *fur* was replaced with the kan^R^-*sacB* cassette. Transformants in which the kan^R^-*sacB* cassette was replaced with the unmarked deletion of *fur* resulting from homologous recombination between the plasmid pKR158 and the chromosome were enriched using sucrose counterselection as described in [42]. Sucrose-resistant isolates were screened for kanamycin sensitivity, and deletion of *fur* in kanamycin-sensitive isolates was confirmed by PCR using the primer pair *fur*_US-F and *fur*_DS_R and DNA sequencing of the resulting amplicons (Eton Biosciences, Research Triangle, NC, USA). For both the wild-type and ΔfapH pflA* backgrounds, two Δ*fur* isolates (strains H177 and H178 for the wild-type background and strains H181 and H182 for the ΔfapH pflA* background) were saved and characterized further.

### 2.4. Motility Assay in Soft Agar Medium

Motility was evaluated using a semisolid medium containing Mueller–Hinton broth, 10% heat-inactivated horse serum, 20 mM 2-(4-morpholino)-ethane sulfonic acid (Sigma-Aldrich, St. Louis, MO, USA) (pH 6.0), and 0.4–0.6% Noble agar (Research Products International, Mt. Prospect, IL, USA). A minimum of three technical replicates were used to assess the motility of each strain. *H. pylori* strains grown on TSA-HS for 2 days were stab-inoculated into the motility agar and incubated at 37 °C under an atmospheric condition consisting of 10% CO_2_, 8% O_2_, and 84% N_2_. The diameters of the resulting swim halos were measured 7 days post-inoculation, and the statistical significance of any differences between strains was determined using a two-sample *t*-test. For motility-base antibiotic sensitivity assays, bacitracin or polymyxin B were included in the soft agar medium. For motility assays that involved the depletion of intracellular iron, a stock solution of 100 mM 2,2′-dipyridyl was prepared in ethanol and added to the motility agar to give a final concentration of 25 µM to 100 µM 2,2′-diyridyl. *H. pylori* strains were grown on TSA-HS for 2 days, then inoculated into the motility agar supplemented with 2,2′-dipyridyl and incubated as described above.

### 2.5. Transmission Electron Microscopy

*H. pylori* strains were grown to late-log phase (OD_600_ ~1.0) in Mueller–Hinton broth supplemented with 5% heat-inactivated horse serum. Cells from 1 mL of culture were collected by centrifugation at 550× *g*, and the resulting cell pellet was resuspended in 125 μL of phosphate-buffered saline (PBS). Quantities of 50 μL of 16% EM-grade formaldehyde (Electron Microscopy Sciences, Hatfield, PA, USA) and 25 μL of 8% EM-grade glutaraldehyde (Electron Microscopy Sciences) were added to the cell resuspension to fix the cells. After incubating at room temperature for 5 min, 10 μL of the cell suspension was applied to a 300-mesh, formvar-coated copper grid and incubated at room temperature for 5 min. Filter paper was used to remove the liquid from the grids. The grids were then washed 3 times with 10 μL of water, removing the water with filter paper after each wash. A quantity of 10 μL of 1% uranyl acetate was applied to the grids for 30 s to stain the cells. After removing the uranyl acetate solution with filter paper, the grids were washed three times as described above and then air-dried. Cells were visualized using a JEOL JEM2100-plus (Thermo Fisher Scientific, Waltham, MA, USA) transmission electron microscope at 120 kv. The number of flagella per cell were determined for at least 100 cells for each strain.

### 2.6. Whole-Genome Sequencing and Analysis

gDNA from the *H. pylori* strains was submitted to the SeqCenter (Pittsburgh, PA, USA) for genomic library preparation and Illumina sequencing. The *breseq* computational pipeline [43] was used to map reads for the *H. pylori* gDNA sequence with the genome for *H. pylori* B128 in the NCBI database (accession no.: NZ_CP024951.1).

## 3. Results

### 3.1. Bacitracin-Resistant Isolates of the ΔfapH pflA* Mutant Have Mutations in fur and hp0771

**Figure 1:**
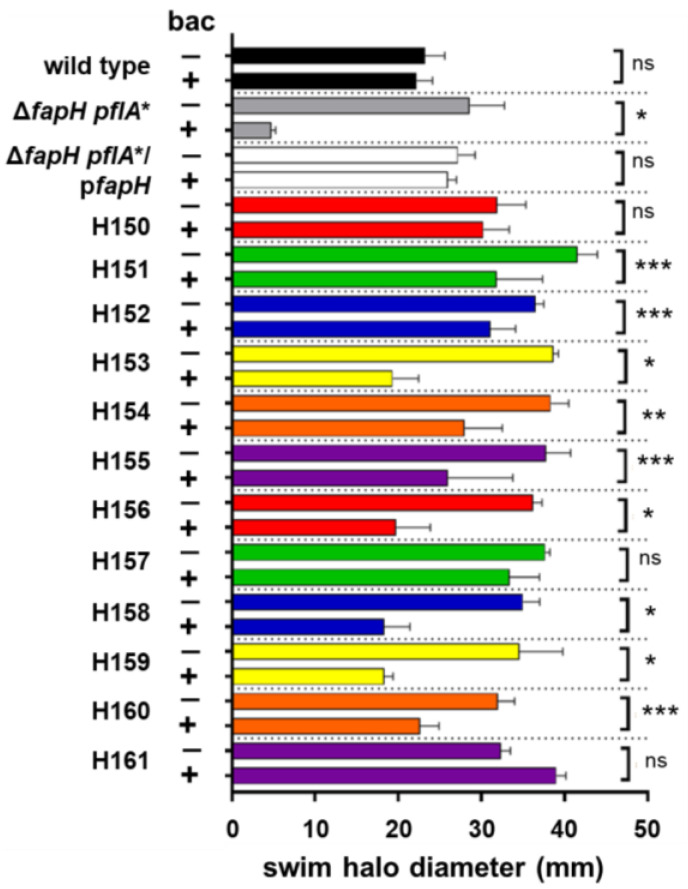
Swim halo formation by bacitracin-resistant isolates of the ΔfapH pflA* mutant in the absence and presence of bacitracin. *H. pylori* strains were inoculated into soft agar medium that lacked (−) or contained (+) 200 μg/mL bacitracin (bac) and then incubated under microaerobic conditions at 37 °C. Diameters of the resulting swim halos were measured 7 days post-inoculation. The ΔfapH pflA* strain failed to form a discernable swim halo but formed a small colony at the point of inoculation. The *H. pylori* strains that were evaluated were the *H. pylori* B128 wild type (wild type), the parental ΔfapH pflA* mutant, the ΔfapH pflA* mutant complemented with a plasmid-borne copy of *fapH* (ΔfapH pflA*/p*fapH*), and the twelve bacitracin-resistant isolates of the ΔfapH pflA* mutant (H150-H161). At least three replicates were performed for each strain. Bars indicate mean values for the diameters of the swim halos and the error bars indicate the standard errors of the means. For each strain, a two-sample *t*-test was used to determine if differences in the sizes of swim halos generated in the absence or presence of bacitracin were statistically significant. One asterisk (*) corresponds to a *p*-value < 0.0001, two asterisks (**) correspond to a *p*-value of <0.001, three asterisks (***) correspond to a *p*-value of <0.05, and ‘ns’ indicates no significant difference.

To investigate the basis of the sensitivity of the ΔfapH pflA* mutant to bacitracin, we enriched for bacitracin-resistant variants of the mutant by repeated passage in soft agar medium containing bacitracin. Following each passage, cells were picked from the edge of the swim halo for inoculation in fresh soft agar medium supplemented with bacitracin. After a few passages, the swim halos formed by the strain were robust, and cells were streaked on solid medium to obtain clonal isolates. Twelve isolates from independent enrichments (designed as strains H150–H161) were examined for their relative resistance to bacitracin by inoculating the strains into soft agar medium that either lacked or contained 200 μg/mL bacitracin, allowing the cells to migrate from the point of inoculation, and then measuring the diameters of the resulting swim halos. This procedure was used to assess the bacitracin resistance of the isolates to avoid enriching for mutations that result in loss of motility, since the bacitracin sensitivity of the ΔfapH pflA* mutant is dependent on functional flagella [28]. The isolates generated significantly larger swim halos in soft agar medium supplemented with bacitracin compared to the parental ΔfapH pflA* strain (Figure 1), suggesting that the isolates are more resistant to bacitracin than the parental strain. As reported previously [28], the sizes of the swim halos generated by wild-type *H. pylori* B128 and the ΔfapH pflA* strain complemented with a plasmid-borne copy of *fapH* (a strain designated as ΔfapH pflA*/p*fapH*) in soft agar medium that either lacked or contained bacitracin were indistinguishable (Figure 1). Including bacitracin in the medium did not reduce the sizes of the swim halos generated by three of the isolates (H150, H157, and H161) but did result in a reduction in the sizes of the swim halos generated by the other isolates (Figure 1), suggesting that the levels of bacitracin resistance differed among the isolates.

Whole-genome sequencing of the bacitracin-resistant isolates revealed that each strain had multiple single-nucleotide polymorphisms (SNPs) when compared to the parental ΔfapH plfA* strain (Table S2). Many of the SNPs in the isolates were within intragenic regions or pseudogenes. Notably, all of the isolates had SNPs within the coding regions of *fur*, which encodes the ferric uptake regulator, and *hp0771*, which encodes an integral membrane protein of unknown function (Table S2). In the case of the *fur* mutations, nine of the isolates (H150, H151, H152, H155, H157, H158, H159, and H161) had an additional adenosine within a homopolymeric run of 8 As that resulted in a frameshift at codon 18 (Table S2). *H. pylori* Fur is 150 amino acid residues in length, and the frameshift in codon 18 results in a severe truncation of the protein. The other three isolates (H154, H156, and H160) had a missense mutation in *fur* that changed Asp-135 to Asn (Table S2). Asp-135 is located within the S3 structural metal-binding site that is occupied by a metal ion in both the Fe-Fur and apo-Fur forms and is suggested to be involved in proper metal ion coordination in apo-Fur [44]. Substitutions in the S3 region of Fur frequently result in a Δ*fur*-like phenotype with regard to the regulation of *amiE*, a gene that is repressed by Fe-Fur [45].

In the case of the SNPs in *hp0771*, seven of the isolates (H150, H151, H152, H155, H157, H159, and H161) had an additional thymidine within a homopolymeric run of 8 Ts that resulted in a frameshift in codon 20 (Table S2). HP0771 has eight predicted transmembrane (TM) helices and is 248 amino acids in length, and the frameshift in codon 20 results in a severe truncation of the protein. The other five isolates (H153, H154, H156, H158, and H160) had a 27 bp in-frame deletion (Table S2) that resulted in the deletion of nine amino acid residues (Leu-21 through Leu-29 within the TM-1 helix (Val-12 through Ser-32)). The 3′-end of the deleted region contains a 10 nt sequence (5′-TTGTTGTTTT-3′) that matches perfectly with the 10 nucleotides that precede the deleted region, which likely contributed to the deletion event via replication slippage or some other mechanism [46]. Table 1 summarizes the relevant genotypes and antibiotic sensitivities of ΔfapH pflA* and the bacitracin-resistant isolates of the strain, as well as other strains generated in the study.

### 3.2. Deletion of Fur Suppresses the Bacitracin Sensitivity of the ΔfapH pflA* Mutant

**Figure 2:**
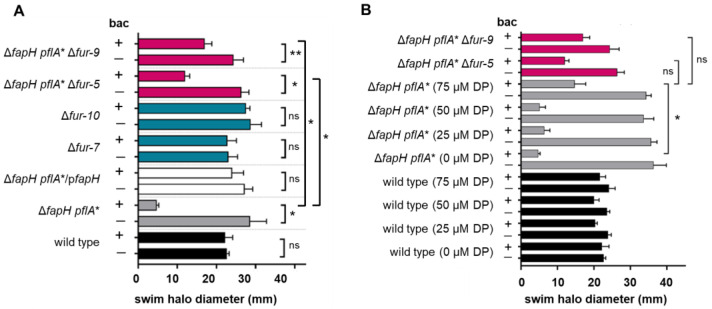
Deletion of *fur* or depletion of intracellular ferrous iron suppresses the bacitracin sensitivity of the ΔfapH pflA* mutant. (**A**) *H. pylori* strains were inoculated into soft agar medium that lacked (−) or contained (+) 200 μg/mL bacitracin (bac) and then incubated under microaerobic conditions at 37 °C. Diameters of the resulting swim halos were measured 7 days post-inoculation. The *H. pylori* strains that were examined were the *H. pylori* B128 wild type (wild type), the ΔfapH pflA* mutant, the ΔfapH pflA* mutant complemented with a plasmid-borne copy of *fapH* (ΔfapH pflA*/p*fapH*), two isolates of the *fur* deletion in the *H. pylori* B128 wild type (Δ*fur-7* and Δ*fur-10*), and two isolates of the *fur* deletion in the ΔfapH pflA* mutant (ΔfapH pflA* Δ*fur-5* and ΔfapH pflA* Δ*fur-9*). (**B**) *H. pylori* strains were inoculated into soft agar medium that lacked or contained 200 μg/mL bacitracin (bac) and various concentrations of 2,2′-dipyridyl (DP), which are indicated within the parentheses (0 μM, 25 M, 50 μM, or 75 μM). After 7 days, the diameters of the resulting swim halos were measured. At least three replicates were performed for each strain and condition. Bars indicate mean values for the diameters of the swim halos and the error bars indicate the standard errors of the means. The bars for the two isolates of the *fur* deletion in the ΔfapH pflA* background are in the same color as the have the same relevant genotype. Similarly, the bars for the two isolates of the *fur* deletion in the wild-type background are in the same color since they have the same relevant genotype. Statistical analysis of the data was performed using a two-sample *t*-test. One asterisk (*) corresponds to a *p*-value < 0.0001, two asterisks (**) correspond to a *p*-value of <0.001, and ‘ns’ indicates no significant difference.

Given that Fur is a global regulator in *H. pylori*, we hypothesized that the mutations in *fur* had a role in suppressing the antibiotic sensitivity in the bacitracin-resistant isolates of the ΔfapH pflA* mutant. To test the validity of the hypothesis, *fur* was deleted in the ΔfapH pflA* mutant and two isolates of the resulting ΔfapH pflA* Δ*fur* strain (designated as ΔfapH pflA* Δ*fur-5* and ΔfapH pflA* Δ*fur-9*) were examined for their motility in soft agar medium and sensitivity to bacitracin. In addition, *fur* was deleted in the *H. pylori* B128 wild type and two isolates of the resulting Δ*fur* mutant (designated as Δ*fur-7* and Δ*fur-10*) were examined for their motility in soft agar medium and sensitivity to bacitracin. Similar to the wild type and the ΔfapH pflA*/p*fapH* strain, swim halo formation by the two isolates of the Δ*fur* mutant was unaffected by the inclusion of bacitracin in the soft agar medium (Figure 2A). Moreover, the isolates of the Δ*fur* mutant displayed wild-type motility in soft agar medium (Figure 2A). Strains ΔfapH pflA* Δ*fur-5* and ΔfapH pflA* Δ*fur-9* generated swim halos in medium containing bacitracin that were significantly larger than the swim halos generated by their parental ΔfapH pflA* strain (Figure 2A), indicating that deletion of *fur* at least partially suppressed the bacitracin sensitivity of the ΔfapH pflA* mutant.

### 3.3. Depletion of Ferrous Iron Suppresses the Bacitracin Sensitivity of the ΔfapH pflA* Mutant

**Figure 3:**
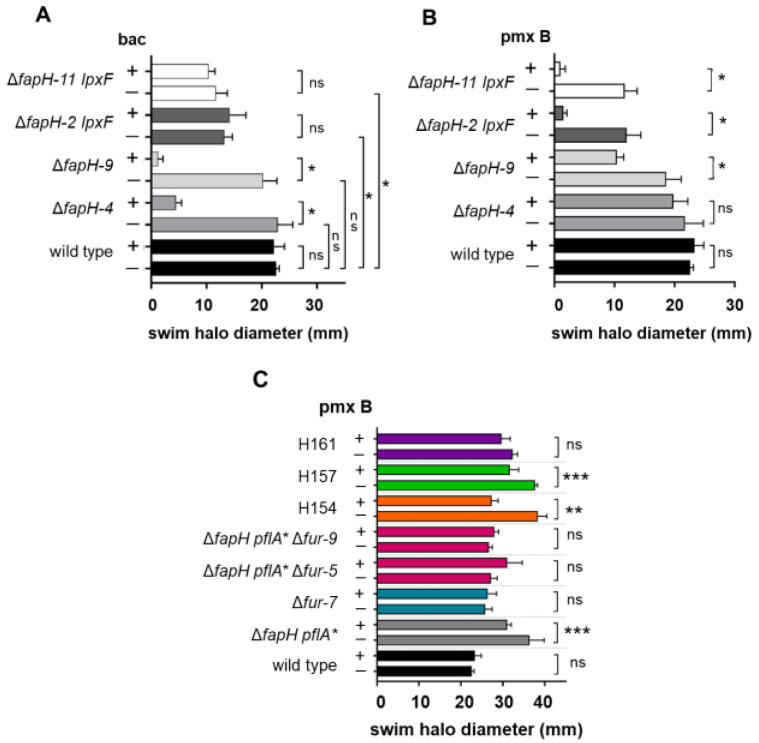
*H. pylori* B128 Δ*fapH* mutants that have a loss-of-function mutation in *lpxF* display resistance to bacitracin. (**A**) Motilities of four *H. pylori* B128 Δ*fapH* isolates (Δ*fapH-4*, Δ*fapH-9*, Δ*fapH-2 lpxF*, and Δ*fapH-11 lpxF*) in soft agar that contained (+) or lacked (−) 200 μg/mL bacitracin (bac) were compared with that of the *H. pylori* B128 wild type. (**B**) Sensitivities of the *H. pylori* B128 wild type and the four *H. pylori* B128 Δ*fapH* isolates to polymyxin B were examined by inoculating the strains into soft agar medium that contained (+) or lacked (−) 3 μg/mL polymyxin B (pmx B) and measuring the diameters of the resulting swim halos 7 days post-inoculation. (**C**) Sensitivities of some of the other *H. pylori* strains used in the study to polymyxin B were examined by inoculating the strains into soft agar medium that contained (+) or lacked (−) 3 μg/mL polymyxin B (pmx B) and measuring the diameters of the resulting swim halos 7 days post-inoculation. At least three replicates were performed for each strain and condition. Bars indicate mean values for the diameters of the swim halos and the error bars indicate the standard errors of the means. The bars for the two isolates of the *fur* deletion in the ΔfapH pflA* background are in the same color as they have the same relevant genotype. For each strain, a two-sample *t*-test was used to determine if differences in the sizes of swim halos generated in the absence or presence of the antibiotic were statistically significant. One asterisk (*) indicates a statistically significant difference (*p*-value < 0.0001), two asterisks (**) correspond to a *p*-value of <0.001, three asterisks (***) correspond to a *p*-value of <0.05, and ‘ns’ indicates no significant difference.

Since three of the bacitracin-resistant isolates of the ΔfapH pflA* mutant expressed a variant of Fur (Fur^D135N^) that is presumably deficient in metal binding, we hypothesized that conditions that favored accumulation of apo-Fur would suppress the bacitracin sensitivity of the ΔfapH pflA* mutant. To address this hypothesis, we deleted the levels of intracellular ferrous iron in the ΔfapH pflA* mutant by the adding 2,2′-dipyridyl to the growth medium and assessing how this impacted the sensitivity of the mutant to bacitracin. 2,2′-dipyridyl is a membrane-permeable ferrous iron chelator that has been used to deplete intracellular iron in bacteria, including *H. pylori* [39,47,48]. Since iron is required for growth of *H. pylori*, we initially examined the growth of wild-type *H. pylori* B128 in soft agar medium that contained concentrations of 2,2′-dipyridyl ranging from 25 μM to 100 μM. *H. pylori* B128 grew poorly in medium containing 100 μM 2,2′-dipyridyl but grew well at lower concentrations of the chelator. Wild-type *H. pylori* B128 formed a robust swim halo in soft agar medium containing bacitracin that was supplemented with 25 μM, 50 μM, or 75 μM 2,2′-dipyridyl (Figure 3B). The ΔfapH pflA* mutant failed to form a swim halo or generated a very small swim halo in soft agar medium containing bacitracin that was supplemented with either 25 μM or 50 uM 2,2′-dipyridyl (Figure 2B). In soft agar medium containing bacitracin that was supplemented with 75 μM 2,2′-dipyridyl, however, the ΔfapH pflA* mutant formed a swim halo that was comparable in size to swim halos formed by the ΔfapH pflA*, Δ*fur-5*, and ΔfapH pflA* Δ*fur-9* strains (Figure 2B). Taken together, these data indicate that culture conditions that favor the accumulation of apo-Fur phenocopy the *fur* deletion in suppressing the bacitracin sensitivity of the ΔfapH pflA* mutant.

### 3.4. H. pylori B128 ΔfapH Isolates That Have a Loss-of-Function Mutation in the LPS Biosynthetic Pathway Gene lpxF Display Resistance to Bacitracin

In a previous study where we characterized the ΔfapH pflA* mutant, we generated additional isolates of the *H. pylori* B128 Δ*fapH* mutant that lacked mutations in *pflA* to confirm that the PflA variant expressed in the ΔfapH pflA* mutant was not responsible for the bacitracin sensitivity of the strain [28]. Two isolates from the reconstruction of the Δ*fapH* mutant (designated as isolates Δ*fapH-2* and Δ*fapH-11*) had reduced motility in soft agar medium compared to the wild type but were not characterized further at that time. As shown in Figure 3A, the motility of Δ*fapH-2* and Δ*fapH-11* in soft agar medium is significantly reduced compared to the wild type and two other isolates from the Δ*fapH* mutant reconstruction (designated as isolates Δ*fapH-4* and Δ*fapH-9*). Interestingly, Δ*fapH-2* and Δ*fapH-11* lacked the sensitivity to bacitracin that was observed with Δ*fapH-4* and Δ*fapH-9* (Figure 3A).

Whole-genome sequencing of the Δ*fapH* isolates confirmed that *fapH* was deleted in all four isolates (Table S2). Both Δ*fapH-2* and Δ*fapH-11* had the same nonsense mutation in *lpxF* that changed codon 14 from TGG (coding for tryptophan) to a TGA stop codon, which effectively results in an *lpxF*-null mutant. While other SNPs were identified in the genomes of Δ*fapH-2* and Δ*fapH-*11, the nonsense mutation in *lpxF* was the only mutation that was shared by the two Δ*fapH* isolates, and the *lpxF* mutation was not identified in Δ*fapH-4* or Δ*fapH-9* (Table S2). LpxF removes the 4′-phosphate from the lipid A backbone and is the final inner membrane component contributing to LPS biosynthesis before the molecule is trafficked to the OM [40]. *H. pylori lpxF* mutants are highly sensitive to the cationic antimicrobial peptide polymyxin B, which presumably results from the presence of the 4′-phosphate on the lipid A backbone that promotes the binding of polymyxin to the cell surface [40]. Polymyxins are small lipopeptides that include a polycationic peptide ring with a short protruding peptide to which a fatty acid tail is attached. Polymyxin B is a mixture of two major polymyxins (B3 and B6) and three minor polymyxins (B1, B1-1, and B2) [49]. While LPS is the initial target for polymyxins, the exact mode of action of these antimicrobial compounds remains unclear [50,51]. The cationic polymyxin peptide ring binds electrostatically to the phosphate residues of lipid A and displaces Mg^2+^ and Ca^2+^ ions that cross-bridge adjacent lipid A molecules and stabilize the OM [51]. Displacement of the divalent metal cations with the bulkier polycationic polymyxin disrupts the barrier function of the OM, allowing for the uptake of previously nonpermeable or weakly permeable molecules and the leakage of periplasmic proteins [52].

**Figure 4:**
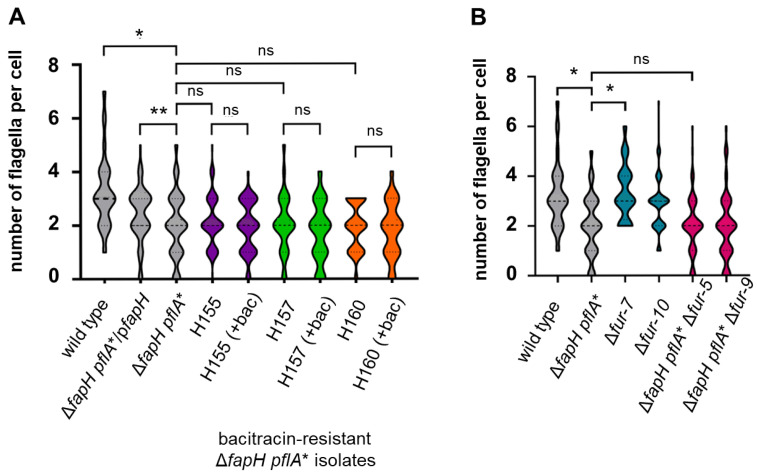
Flagellar numbers for *H. pylori* strains harvested from soft agar medium. (**A**) Violin plots of the data for the number of flagella per cell for wild-type *H. pylori* B128, the ΔfapH pflA* mutant, the ΔfapH pflA* mutant complemented with a plasmid-borne copy of *fapH* (ΔfapH pflA*/p*fapH*), and the bacitracin-resistant ΔfapH pflA* isolates H155, H157, and H160. (**B**) Violin plots of the data for the number of flagella per cell for wild-type *H. pylori* B128, the ΔfapH pflA* mutant, the Δ*fur* mutant against the wild-type background (Δ*fur-7* and Δ*fur-10*), and the Δ*fur* mutants in the ΔfapH pflA* background (ΔfapH pflA* Δ*fur-5* and ΔfapH pflA* Δ*fur-9*). Flagellar counts were performed for at least 100 cells for each strain. Mean values for the number of flagella per cell for each strain are indicated within the violin plots and were as follows: wild type (3.25), ΔfapH pflA* (2.03), ΔfapH pflA*/p*fapH* (2.39), H155 (2.14), H157 (2.04), H160 (1.96), Δ*fur-7* (3.25), Δ*fur-10* (2.84), ΔfapH pflA* Δ*fur-5* (1.96), and ΔfapH pflA* Δ*fur-9* (1.93)—mean values are indicated within the parentheses. Statistical significance was determined by a one-way ANOVA with multiple comparisons. The asterisk indicates a significant difference with a *p*-value < 0.0001, and two asterisks (**) correspond to a *p*-value of <0.001.

As expected, growth of the Δ*fapH-2* and Δ*fapH-11* isolates in soft agar medium containing 3 μg/mL polymyxin B was severely impaired (Figure 3B), which confirmed that the *lpxF* mutations in these strains resulted in loss of function of the gene. In contrast, wild-type *H. pylori* B128 and one of the other Δ*fapH* isolates (Δ*fapH-4*) exhibited no growth defect in the presence of 3 μg/mL polymyxin B (Figure 3B). The other Δ*fapH* isolate (Δ*fapH-9*) displayed some sensitivity to polymyxin B, but in contrast to Δ*fapH-2* and Δ*fapH-11*, it formed a robust swim halo in the presence of polymyxin B (Figure 3B). The Δ*fapH-9* isolate appeared to be more sensitive to bacitracin than Δ*fapH-4* (Figure 4A), which suggests that the barrier function of the OM is more impaired in Δ*fapH-9* and may have accounted for the greater sensitivity of the isolate to polymyxin B. The Δ*fapH-9* isolate had a mutation, *hp0839* (encoding a homolog of the *E. coli* FadL fatty acid transport protein) (Table S2), which may have accounted for the increased antibiotic sensitivity of Δ*fapH-9* compared to Δ*fapH-4*. The *hp0839* allele in Δ*fapH-9* had a 30 bp sequence that was replaced with a 27 bp sequence of unknown origin, which altered the sequence of Asn-165 through Thr-175 of the protein (sequence altered from N^165^PDTQIVNGWT^175^ to I^165^EFPRPPWRP^174^). *E. coli* FadL is a β-barrel protein that transports exogenous fatty acids across the OM [53]. FadL is proposed to be part of a signaling process that increases LPS biosynthesis in response to fatty acids released by the phospholipase PldA from phospholipids that mislocalize in the outer leaflet of the OM [54]. It is possible that, as with *E. coli* FadL, HP0839 has a role in the maintenance of OM homeostasis and that the mutation in *hp0839* interfered with this role and further compromised the barrier function of the OM in the absence of FapH.

The alterations to the lipid A backbone in the absence of LpxF strengthen the OM by allowing Mg^2+^ and Ca^2+^ ions to cross-bridge adjacent lipid A molecules and increasing hydrophobic interactions in the lipid bilayer due to the additional acyl chains. We hypothesize that loss of LpxF suppressed the bacitracin sensitivity of the Δ*fapH* isolates by strengthening the OM to limit diffusion of the antibiotic into the periplasmic space. To determine if changes in the expression or activity of LpxF may have accounted for the suppression of the bacitracin sensitivity in the bacitracin-resistant isolates of the ΔfapH pflA* mutant, we examined the sensitivity of the strains to polymyxin B. In addition, we examined the sensitivity of the Δ*fur* mutants to polymyxin B. While growth of some of the bacitracin-resistant isolates was inhibited slightly by polymyxin B, none of the strains displayed the severe sensitivity to polymyxin B observed with the Δ*fapH* strains that had the nonsense mutation in *lpxF* (Figure 3C; all 12 bacitracin-resistant isolates were examined but results are only shown for three of the isolates). Similarly, the strains in which *fur* was deleted appeared to retain resistance to polymyxin B (Figure 3C). These findings suggest that the suppression of the bacitracin sensitivity in the bacitracin-resistant isolates of the ΔfapH pflA* and the ΔfapH pflA* strains in which *fur* had been deleted was not due to loss of LpxF activity.

### 3.5. Cells of the ΔfapH pflA* Mutant Possess Fewer Flagella When Cultured in Soft Agar Medium

Transcriptome analysis of a *H. pylori* G27 *fur* knockout mutant identified several flagellar genes (*flaB*, *flgL*, *fliY*, and *fliY*) that were down-regulated in the mutant [37]. Based on this previous observation, we hypothesized that loss of Fur activity may reduce the number of flagella per cell in *H. pylori*, which may have accounted for the increased antibiotic resistance of the ΔfapH pflA* bacitracin-resistant isolates. In other words, we reasoned that ΔfapH pflA* cells that have fewer flagella are likely to incur less damage to the OM and therefore display increased resistance to bacitracin. To examine the validity of the hypothesis, we examined cells of the three ΔfapH pflA* bacitracin-resistant isolates (H155, H157, and H160) by transmission electron microscopy (TEM) to quantify the number of flagella per cell. These strains were examined since two of them have the frameshift mutation in *fur* (H155 and H157), while the other strain expresses the FurD^135N^ variant (H160). For the analysis, we examined cells cultured in soft agar medium to duplicate the conditions used to assess the strains’ sensitivity to bacitracin. Wild-type *H. pylori* B128 cells displayed an average of 3.25 flagella per cell, while the number of flagella per cell was significantly lower for the ΔfapH pflA* mutant (mean = 2.03) (Figure 4). Notably, there were a greater number of ΔfapH pflA* cells that had either no flagella or a single flagellum compared to the wild type. This result was somewhat surprising, since we had observed previously that there was no difference between the wild type and the ΔfapH pflA* mutant in the flagellar number for cells grown in liquid medium [28]. Complementation of the ΔfapH pflA* mutant with a plasmid-borne copy of *fapH* resulted in a slight, but statistically significant, increase in the number of flagella per cell (mean = 2.39) (Figure 4A). The results with the complemented strain suggest that loss of FapH is at least partially responsible for the reduced flagellation of the ΔfapH pflA* mutant, but another genetic determinant may play a role in the altered flagellation pattern of the mutant. The flagellation patterns of the cell populations (i.e., the average number of flagella per cell and the distribution of flagellar numbers) of the bacitracin-resistant ΔfapH pflA* isolates were indistinguishable from that of the parental ΔfapH pflA* mutant (Figure 4A). Including bacitracin in the soft agar medium did not affect the flagellation patterns of the bacitracin-resistant ΔfapH pflA* isolates (Figure 4A). Taken together, these findings indicate that the antibiotic resistance in the bacitracin-resistant ΔfapH pflA* isolates does not result from the cells having fewer flagella, nor does including bacitracin in the soft agar medium enrich for cells that have fewer flagella.

**Figure 5:**
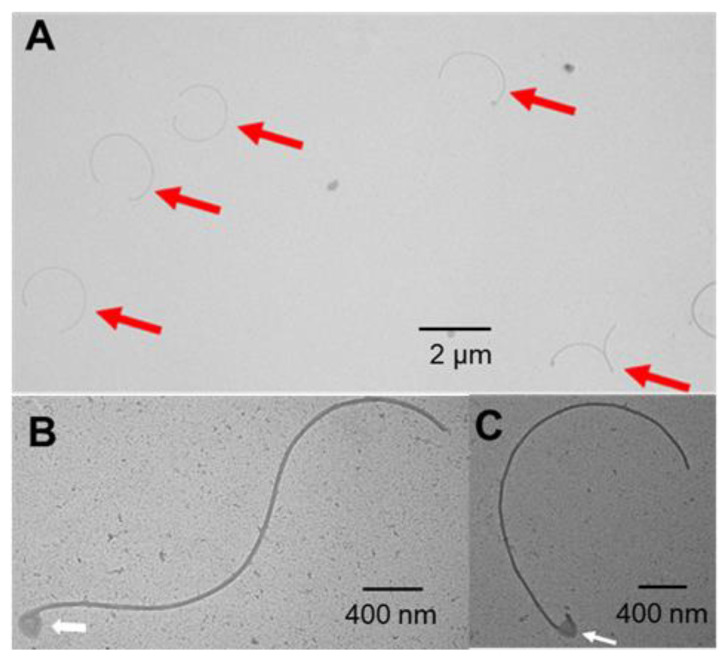
TEM images of detached flagella. (**A**) TEM field for sample of the ΔfapH pflA* mutant that has several detached flagella that are indicated by the red arrows. (**B**,**C**) Higher magnification of detached flagella that have possible hooks and associated membranous material that are indicated by the white arrows.

We investigated further whether the loss of *fur* affected flagellation in the wild-type *H. pylori* B128 background by examining cells of the Δ*fur-7* and Δ*fur-10* mutants harvested from the soft agar medium. The flagellation patterns of the two Δ*fur* mutants were indistinguishable from that of the parental wild-type strain (Figure 5B). As expected given the results for bacitracin-resistant ΔfapH pflA* isolates, the flagellation patterns of the ΔfapH pflA* strains in which *fur* was deleted were the same as that of the parental Δ*fapH* ΔpflA* mutant (Figure 4B). In all four of the *fur* deletion mutants, the flagellation phenotypes of the mutants were the same as that of the parental strain, indicating that the loss of Fur has no impact on the flagellation pattern in *H. pylori*.

During the course of the study quantifying the flagellar numbers for the various strains, we often observed detached flagella in samples of the ΔfapH pflA* mutant (Figure 5). The presence of the detached flagella was readily apparent, and we frequently observed several flagella within a TEM field (Figure 5A). In contrast, detached flagella in samples of wild-type *H. pylori* B128 were rarely observed. In many of the detached flagella from the ΔfapH pflA* mutant, one of the ends was bent sharply and appeared to be part of the hook (Figure 5B,C). The hook-like structures typically had associated material that may have been remnants of the sheath or OM. It is possible that the flagella of ΔfapH pflA* mutants were more prone to be sheared from the bacteria during the preparation of the samples for TEM. Alternatively, the flagella may have been shed by the bacteria as they were growing in the soft agar medium. Regardless of the reason for the prevalence of detached flagella in the samples of the ΔfapH pflA* mutant, the detachment of the flagella likely accounts for the reduced number of flagella associated with the mutant.

## 4. Discussion

The FapH-ring is a *H. pylori* motor accessory that is associated with the OM and is proposed to protect the barrier function of the OM from damage resulting from the high-speed rotation of the polar, sheathed flagella of the bacterium [28]. We report here on the further characterization of FapH and its role in protecting the OM from flagellum-mediated damage by characterizing 12 independently isolated bacitracin-resistant variants of the ΔfapH pflA* mutant (Figure 1). Whole-genome sequencing of the bacitracin-resistant isolates revealed that all of the isolates had mutations in *fur* and *hp0771* (Table S2). Two types of mutations in *fur* were identified in the bacitracin-resistant ΔfapH pflA* isolates. One of the mutations was a frameshift that occurred early in the coding region of the gene and essentially resulted in a *fur* null mutation. The second type of *fur* mutation was a missense mutation that altered Asp-135, which is located in one of the metal-binding sites of Fur [44], to asparagine. Deletion of *fur* in the ΔfapH pflA* mutant partially suppressed the bacitracin sensitivity of the strain (Figure 2A). We further showed that the sensitivity of the ΔfapH pflA* mutant to bacitracin was partially suppressed by depleting intracellular ferrous iron in the strain by the addition of 2,2′-dipyridyl to the growth medium (Figure 2B). Taken together, these results indicate that loss of Fur or growth conditions that favor the accumulation of apo-Fur mitigate damage to the barrier function of the OM that results from flagellar rotation in the ΔfapH pflA* mutant.

We postulate that the altered expression of one or multiple Fur-regulated genes resulting from the deletion of *fur* or the depletion of intracellular ferrous iron suppresses the bacitracin sensitivity of the ΔfapH pflA* mutant. There are several Fur-regulated genes whose altered expression might be responsible for suppressing the bacitracin sensitivity of the ΔfapH pflA* mutant that are reasonable candidates for such a role. Two such candidates are the LPS biosynthesis genes, *wecA* (*hp1581*) and *hp0826*, which were modestly up-regulated in a *H. pylori* G27 *fur* knockout mutant [37]. WecA is a glycosyltransferase that initiates the assembly of the O polysaccharide on the undecaprenyl phosphate carrier in LPS biosynthesis [55], while HP0826 is a glycosyltransferase involved in chain elongation of the type 2 Lewis antigen of LPS [56]. It is possible that the increased expression of *wecA* and/or *hp0826* in the absence of Fur affects the length of the O antigen and strengthens the OM to mitigate flagellum-mediated damage to the OM in the absence of FapH. Alternatively, genes encoding several OM proteins involved in iron transport (*fecA-1*, *fecA-2*, and *frpB-1*) are drastically up-regulated in the absence of Fur [37,57]. It may be that high levels of these OM proteins stabilize the OM in the ΔfapH pflA* mutant.

Roier and co-workers reported on a link between Fur and the maintenance of lipid asymmetry in the OM in *E. coli*, *Vibrio cholerae*, and *Haemophilus influenzae* [58]. *H. influenzae* subjected to iron limitation by including 2,2′-dipyridyl in the growth medium produced more OMVs compared to *H. influenzae* grown under iron-replete conditions, and transcript levels of *vacJ* and *yrbE* (genes involved in the maintenance of lipid asymmetry in the OM) were ~2-fold lower in *H. influenzae* grown under the iron-limiting condition [58]. In addition, a *H. influenzae* Δ*fur* mutant grown under iron-replete conditions produced more OMVs, and expression of *vacJ* and *yrbE* in the Δ*fur* mutant was reduced ~10-fold and ~2-fold, respectively [58]. Similar to the observations in *H. influenzae*, Δ*fur* mutants of *V. cholerae* and *E. coli* displayed increased OMV production and decreased expression of *vacJ*/*mlaA* and *yrbE*/*mlaE* [58]. We do not believe that the deletion of *fur* suppressed the bacitracin sensitivity of the ΔfapH pflA* mutant by decreasing expression of the maintenance of lipid asymmetry (Mla) pathway genes, since such an outcome would be expected to exacerbate the sensitivity of the mutant to bacitracin. Moreover, the expression of the genes encoding the *H. pylori* Mla homologs (HP1463, HP1464, HP1465, and HP1466 [58]) was not reported to be affected by deletion of *fur* [37,57].

Given that all of the bacitracin-resistant isolates of the ΔfapH pflA* mutant had a mutation in *hp0771*, it seems likely that disrupting the function of HP0771 has a role in suppressing the bacitracin sensitivity of the mutant. Two types of mutations in *hp0771* were identified in the bacitracin-resistant ΔfapH pflA* isolates. One of the mutations was a frameshift that occurred early in the coding region of the gene and essentially resulted in an *hp0771* null mutation. The second type of mutation was an in-frame deletion that is predicted to result in the loss of nine amino acids in the first TM helix of the protein. It is possible that the combination of mutations in *fur* and *hp0771* are needed to fully suppress the bacitracin sensitivity of the ΔfapH pflA* mutant. HP0771 is an integral membrane protein of unknown function and is encoded in an operon with *amiA*, which encodes a peptidoglycan hydrolase that is required for the morphological transition of *H. pylori* cells from the bacillary to coccoid form [59]. Despite its close linkage with *amiA*, *hp0771* is not required for the normal morphological transition from the bacillary to the coccoid form [59]. Future studies should determine if loss of HP0771 has a role in suppressing the bacitracin sensitivity of the ΔfapH pflA* mutant.

In reconstructing the *H. pylori* B128 Δ*fapH* mutant to eliminate secondary mutations in *pflA*, we observed that two of the Δ*fapH* isolates (Δ*fapH-2* and Δ*fapH-11*) were significantly more resistant to bacitracin than the ΔfapH pflA* mutant or the other Δ*fapH* isolates (Figure 3A). The Δ*fapH-2* and Δ*fapH-11* isolates had the same nonsense mutation at codon 14 in *lpxF*, which encodes a phosphatase that removes the 4′-phosphate from the lipid A backbone of LPS [40]. *H. pylori* Δ*lpxF* mutants are highly sensitive to polymyxin B due to the presence of the 4′-phosphate on the lipid A backbone, which facilitates binding of polymyxin B to the LPS molecule [40]. The Δ*fapH-2* and Δ*fapH-11* isolates were very sensitive to polymyxin B (Figure 3B), which verified that LpxF was indeed inactive in the two isolates. In the final step of the LPS biosynthetic pathway in *H. pylori*, LpxR removes a phosphatidic acid from the lipid A backbone to generate a tetra-acylated product [41]. The LPS pathway intermediate that has the 4′-phosphate on lipid A is not recognized efficiently by LpxR, and so the lipid A backbone of LPS in the *H. pylori lpxF* mutant is both phosphorylated at the 4′-position and hexa-acylated [40,41]. The 4′-phosphate on the lipid A backbone allows the molecule to bind divalent metal cations such as Mg^2+^ and Ca^2+^, which mediate interactions with neighboring lipid A molecules and enhance the stability of the OM [60]. Moreover, the additional acyl chains in the lipid A backbone of the *lpxF* mutant presumably increase hydrophobic interactions in the lipid bilayer to strengthen further the OM. We hypothesize that these membrane-stabilizing effects compensate for the loss of FapH and minimize damage to the OM during flagellar rotation. Future studies can address the validity of this hypothesis by determining if deleting *lpxF* in the ΔfapH pflA* mutant suppresses the bacitracin sensitivity of the mutant, as well as determine if loss of LpxF results in reduced motility in soft agar medium.

The Δ*fapH-2* and Δ*fapH-11* isolates generated smaller swim halos in the soft agar that were significantly smaller than those produced by the ΔfapH pflA* mutant or the two other Δ*fapH* isolates (Figure 3A,B). Several physiological factors influence swim halo development, including swimming behavior, chemotaxis, growth rate, and microcolony formation. Given that the nonsense mutation in *lpxF* was the only mutation in the Δ*fapH-2* and Δ*fapH-11* isolates that was not found in the ΔfapH pflA* mutant or the two other Δ*fapH* isolates (Table S2), it seems likely that the loss of LpxF was responsible for the smaller swim halos of these strains. Of the physiological factors listed above that influence the migration of *H. pylori* in soft agar, a role for LpxF in microcolony formation seems the most plausible factor. There is precedence for mutations in *H. pylori* affecting migration in soft agar medium by influencing microcolony formation. Loss of PilO or PilN, which form part of a cage-like structure that surrounds each stator unit in the *H. pylori* flagellar motor, enhances swim halo development as a result of continued swimming rather than aggregation into microcolonies [26]. Given that the bacitracin-resistance isolates of the ΔfapH pflA* mutant were obtained by picking bacteria from the edges of the swim halos, the reduced migration of Δ*fapH-2* and Δ*fapH-11* in soft agar medium may have accounted for why we did not obtain any isolates with a mutation in *lpxF* from the enrichment.

Finally, we observed that cells of the ΔfapH pflA* mutant that were harvested from soft agar medium had fewer flagella compared to wild-type cells collected under the same conditions (Figure 4). This result was unexpected, since we had previously observed no difference between the wild type and the ΔfapH pflA* mutant with regard to the number of flagella per cell when the strains were grown in liquid medium [28]. Detached flagella were often observed in the TEM fields of the ΔfapH pflA* mutant (Figure 5), whereas we rarely observed detached flagella associated with wild-type cells. We infer from these observations that the detachment of the flagella accounted for the reduced flagellar number of the ΔfapH pflA* mutant when grown in soft agar medium. It is possible that the flagella of the ΔfapH pflA* mutant are more fragile than the flagella of the wild type and were more likely to be sheared from the bacteria during the preparation of the grids for TEM. We think it more likely, though, that the detached flagella associated with the ΔfapH pflA* mutant were shed by the bacteria as they were cultured in the soft agar medium. The ejection of flagellar filaments is a widespread phenomenon among bacterial species. Some bacteria, such as *Caulobacter crescentus*, have a programmed pathway whereby the bacterium ejects the flagellum upon encountering a surface in order to elaborate an adhesive stalk for surface attachment [61]. Ferreira and co-workers noted that the ejection of flagella at the base of the flagellar hook in response to nutrient deprivation was a common event in multiple species of polar flagellated bacteria [62]. Zhu and co-workers reported that a *Pseudomonas aeruginosa* Δ*fleN* mutant ejected flagella at a significantly higher frequency compared to the wild-type parental strain [63]. FleN regulates flagellar number in *P. aeruginosa* [64], and the *P. aeruginosa* Δ*fleN* mutant assembles multiple, polar flagella, whereas the wild type has a single polar flagellum. Zhu and co-workers speculated that the shedding of flagella by the *P. aeruginosa* Δ*fleN* mutant resulted from the entanglement of the flagella [63]. A relic of the flagellar motor that consists of the LP-ring complex and associated motor accessories is left following the ejection of the flagellar filament [62,63,65]. A plug protein is associated with the flagellar motor relic and is thought to prevent leakage across the OM [62,63,65]. We hypothesize that the ΔfapH plfA* mutant ejected flagella in response to the high viscosity of the soft agar medium. If that hypothesis is valid, then we would expect a high proportion of the ΔfapH pflA* mutant cells grown in viscous medium to possess relics of flagellar motors.

The loss of FapH appeared to be at least partially responsible for the reduced flagellation of the ΔfapH pflA* mutant, since introducing a plasmid-borne copy of *fapH* into the strain resulted in a slight, but significant, increase in the number of flagella per cell (Figure 4A). The failure of the plasmid-borne copy of *fapH* to restore the flagellar number of the ΔfapH pflA* mutant to wild-type levels suggests that another mutation contributed to the decreased flagellation of the strain. From the SNPs identified in the ΔfapH pflA* mutant (Table S2), there are no obvious candidates for a mutation that may have contributed to the decreased flagellation of the strain. It is possible that the pflA* allele contributed to flagellar ejection in the ΔfapH pflA* mutant, which is a hypothesis that can be addressed in future studies.

## Figures and Tables

**Table 1:** Relevant genotypes and phenotypes of *H. pylori* strains examined in the study.

Strain	Description/Relevant Genotype	^a^ Bacitracin Resistance	Polymyxin B Resistance
*H. pylori* B128 wild type	Wild type	+	+
ΔfapH pflA*	Δ*fapH*, *pflA* allele encodes a variant of PflA that has altered amino acid sequence from Leu-465 to Ile-476	−	+
H150	Bacitracin-resistant isolate of ΔfapH pflA*, frameshift in *fur* (codon 18), frameshift in *hp0771* (codon 19)	+	+
H151	Bacitracin-resistant isolate of ΔfapH pflA*, frameshift in *fur* (codon 18), frameshift in *hp0771* (codon 19)	+/−	+
H152	Bacitracin-resistant isolate of ΔfapH pflA*, frameshift in *fur* (codon 18), frameshift in *hp0771* (codon 19)	+/−	+
H153	Bacitracin-resistant isolate of ΔfapH pflA*, frameshift in *fur* (codon 18), 27-bp deletion in *hp0771*	+/−	+
H154	Bacitracin-resistant isolate of ΔfapH pflA*, missense mutation in *fur* (Asp135Asn), 27-bp deletion in *hp0771*	+/−	+
H155	Bacitracin-resistant isolate of ΔfapH pflA*, frameshift in *fur* (codon 18), frameshift in *hp0771* (codon 19)	+/−	+
H156	Bacitracin-resistant isolate of ΔfapH pflA*, missense mutation in *fur* (Asp135Asn), 27-bp deletion in *hp0771*	+/−	+
H157	Bacitracin-resistant isolate of ΔfapH pflA*, frameshift in *fur* (codon 18), frameshift in *hp0771* (codon 19)	+	+
H158	Bacitracin-resistant isolate of ΔfapH pflA*, frameshift in *fur* (codon 18), 27-bp deletion in *hp0771*	+/−	+
H159	Bacitracin-resistant isolate of ΔfapH pflA*, frameshift in *fur* (codon 18), frameshift in *hp0771* (codon 19)	+/−	+
H160	Bacitracin-resistant isolate of ΔfapH pflA*, missense mutation in *fur* (Asp135Asn), 27-bp deletion in *hp0771*	+/−	+
H161	Bacitracin-resistant isolate of ΔfapH pflA*, frameshift in *fur* (codon 18), frameshift in *hp0771* (codon 19)	+	+
ΔfapH pflA* Δ*fur-5*	Deletion of *fur* in ΔfapH pflA* strain	+/−	+
ΔfapH pflA* Δ*fur-9*	Deletion of *fur* in ΔfapH pflA* strain	+/−	+
Δ*fur-7*	Deletion of *fur* in *H. pylori* B128	+	+
Δ*fur-10*	Deletion of *fur* in *H. pylori* B128	+	n.d.
Δ*fapH-2*	Deletion of *fapH* in *H. pylori* B128, nonsense mutation in *lpxF* (codon 14)	+	−
Δ*fapH-11*	Deletion of *fapH* in *H. pylori* B128, nonsense mutation in *lpxF* (codon 14)	+	−
Δ*fapH-4*	Deletion of *fapH* in *H. pylori* B1218	−	+
Δ*fapH-9*	Deletion of *fapH* in *H. pylori* B128	−	+

^a^ Plus sign (+) indicates wild-type level of resistance to 200 µg/mL bacitracin, minus sign (−) indicates the lack of a discernable swim halo in the presence of 200 µg/mL bacitracin, and the plus/minus sign (+/−) indicates the formation of a swim halo that is significantly smaller than that formed by the wild type in the presence of 200 µg/mL bacitracin.

## Data Availability

The original contributions presented in the study are included in the article and Supplementary Materials; further inquiries can be directed to the corresponding author.

## Supplementary Materials

The following supporting information can be downloaded at: https://www.mdpi.com/article/10.3390/microorganisms13092103/s1, Table S1: *H. pylori* strains and plasmids used in the study; Table S2: SNPs identified in *H. pylori* strains used in the study.

## Abbreviations

The following abbreviations are used in this manuscript:

OM	Outer membrane
OMV	Outer membrane vesicle
LPS	Lipopolysaccharide
TSA-HS	Tryptic soy agar supplemented with horse serum
gDNA	Genomic DNA
TEM	Transmission electron microscopy
PBS	Phosphate-buffered saline
LB	Lysogeny broth
SNP	Single-nucleotide polymorphism
TM	Transmembrane
bac	Bacitracin
DP	2,2′-dipyridyl
pmxB	Polymyxin B
