# Supplementary material for: Mutations in the Ferric Uptake Regulator Gene (fur) Suppress the Bacitracin Sensitivity of a Helicobacter pylori fapH Deletion Mutant
Source: Microorganisms. 2025 Sep 9;13(9):2103. doi: 10.3390/microorganisms13092103 (PMC12472139; doi:10.3390/microorganisms13092103)
Supplement: Supplementary file 1 [file microorganisms-13-02103-s001.zip › Table S2_identified SNPs.pdf]

Table S2. SNPs identified in *H. pylori* strains used in the study.

***H. pylori* B128 Δ*fapH pflA** * (lab strain H54) – SNPs not present in wild-type *H. pylori* B128**					
**NCBI designation**	**26695 locus tag**	**Gene/description**	**Mutation/annotation**	[a] **Impact**	[b] **Frequency**
CV725_RS00010	HP1527	*comH*, competence protein	Δ1 bp (708/1440 nt)	Val236fs	98.8%
CV725_RS00395	HP1451	Cag VirB11 inhibitory protein	+T (689/798 nt)	Tyr230fs	98.1%
CV725_RS00960	HP1274	*pflA*, paralyzed flagella protein	+G (1428/2406 nt)<br>Δ1 bp (1391/2406 nt)	offsetting fs<br>mutations	98.4%<br>98.5%
maf<br>*CV725_RS01150*	HP1239<br>HP1240	[c] DS of *maf*, [c] US of *hp1239* (hypothetical protein)	(A)$_{13\rightarrow14}$<br>intergenic (+61/+394)	unknown	80.5%
mreC	HP1372	*mreC*, rod shape-determining protein	codon-220 (TAC→CAC)	Tyr220His	98.8%
CV725_RS02745	HP0098	*thrC*, threonine synthase	codon-417 (GAA→AAA)	Glu417Lys	98.9%
tlpB	HP0103	*tlpB,* methyl-accepting chemotaxis protein	codon-26<br>(GGG→GAG)	Gly26Glu	98.6%
CV725_RS03745	HP0298	*dppA*, dipeptide ABC transporter, periplasmic dipeptide-binding protein	(T)$_{10\rightarrow9}$ (30/1644 nt)	Phe10fs	96.4%
CV725_RS04335	HP1028	hypothetical protein	codon-16<br>(GCT→GTT)	Ala16Val	98.5%
CV725_RS04505	HP0475	*modD*, molybdenum ABC transport protein	codon-155 (TTA→TTG)	Leu155Leu	99.1%
CV725_RS04600<br>*CV725_RS04605*	HP0875<br>HP0876	US of *katA,* US of *frpB*	(A)$_{5\rightarrow6}$<br>intergenic (-34/-290)	unknown	83.4%
CV725_RS04785	HP0838	*fapH*, flagellum-associated protein H	codon-18 (GTC→GCC)<br>Δ490 bp	Val18Ala<br>gene deletion	99.1%
CV725_RS06350	HP0527	cag pathogenicity island protein Cag7	codon-752<br>(ACC→ACG)<br>codon-742<br>(GCT→GCC)	Thr752Thr<br><br>Ala742Ala	96.2%<br><br>99.3%
babA<br>*CV725_RS06645*	HP1243<br>tRNA_fMet	US of *babA*, DS of tRNA_fMet	(T)$_{13\rightarrow12}$<br>intergenic (-131/+149)	unknown	86.2%
CV725_RS08550<br>*queF*	HP1412<br>HP1413	DS of *hp1412* (hypothetical protein), DS of *queF*	(C)$_{11\rightarrow10}$<br>intergenic (+11/+121)	unknown	94.6%
**bacitracin-resistant isolate of Δ*fapH pflA** strain (H150) – SNPs not present in Δ*fapH pflA** parental strain**					
**NCBI designation**	**26695 locus tag**	**Gene/description**	**Mutation/annotation**	**Impact**	**Frequency**
CV725_RS00040	NA	site-specific DNA-methyltransferase, pseudogene	(G)$_{14\rightarrow12}$<br>(1452/2053 nt)	no effect	94.8%
CV725_RS00720<br>*CV725_RS00725*	HP1321<br>HP1322	DS of HP1321 conserved hypothetical ATP-binding protein and US of HP1322 hypothetical protein.	(ATACATAA)$_{10\rightarrow5}$<br>intergenic (-194/+7)	unknown	99.2%
CV725_RS08420	HP1353	Type ISP C-terminal specificity domain, pseudogene	(C)$_{14\rightarrow13}$<br>(3223/3372 nt)	no effect	93.4%
CV725_RS03925	HP0337	hypothetical protein	(A)$_{7\rightarrow8}$ (62/339 nt)	Asn19-fs	98%
fur	HP1027	*fur*, ferric uptake regulator	(A)$_{8\rightarrow9}$ (55/453 nt)	Ile18-fs	97.4%
CV725_RS05120	HP0771	hypothetical protein.	(T)$_{8\rightarrow9}$ (59/738 nt)	Phe19-fs	96.5%
**bacitracin-resistant isolate of Δ*fapH pflA** strain (H151) – SNPs not present in Δ*fapH pflA** parental strain**					

NCBI designation	26695 locus tag	Gene/description	Mutation/annotation	Impact	Frequency
CV725_RS00040	NA	site-specific DNA-methyltransferase, pseudogene	(G)$_{14\rightarrow13}$ (1453/2053 nt)	phase ‘off’ to phase ’on’	91.4%
CV725_RS00720 *CV725_RS00725*	HP1321 HP1322	DS of HP1321 conserved hypothetical ATP-binding protein and US of HP1322 hypothetical protein.	(ATACATAA)$_{10\rightarrow5}$ intergenic (-194/+7)	unknown	100%
CV725_RS08420	HP1353	Type ISP C-terminal specificity domain, pseudogene	(C)$_{14\rightarrow13}$ (3223/3372 nt)	no effect	88.6%
fur	HP1027	*fur*, ferric uptake regulator	(A)$_{8\rightarrow9}$ (55/453 nt)	Ile18-fs	97.1%
CV725_RS05120	HP0771	hypothetical protein.	(T)$_{8\rightarrow9}$ (59/738 nt)	Phe19-fs	97.2%
**bacitracin-resistant isolate of Δ*fapH pflA** strain (H152) – SNPs not present in Δ*fapH pflA** parental strain**					
**NCBI designation**	**26695 locus tag**	**Gene/description**	**Mutation/annotation**	**Impact**	**Frequency**
CV725_RS00040	NA	site-specific DNA-methyltransferase, pseudogene	(G)$_{14\rightarrow13}$ (1453/2053 nt)	phase ‘off’ to phase ’on’	90.3%
CV725_RS00720 *CV725_RS00725*	HP1321 HP1322	DS of HP1321 conserved hypothetical ATP-binding protein and US of HP1322 hypothetical protein.	(ATACATAA)$_{10\rightarrow5}$ intergenic (-194/+7)	unknown	99.6%
CV725_RS08420	HP1353	Type ISP C-terminal specificity domain, pseudogene	(C)$_{14\rightarrow13}$ (3223/3372 nt)	no effect	91.9%
fur	HP1027	*fur*, ferric uptake regulator	(A)$_{8\rightarrow9}$ (55/453 nt)	Ile18-fs	96.7%
CV725_RS05120	HP0771	hypothetical protein.	(T)$_{8\rightarrow9}$ (59/738 nt)	Phe19-fs	96%
hefC	HP0607	efflux RND transporter permease subunit HefC.	codon-808 (GCT→GTT)	Ala808Val	99.5%
**bacitracin-resistant isolate of Δ*fapH pflA** strain (H153) – SNPs not present in Δ*fapH pflA** parental strain**					
**NCBI designation**	**26695 locus tag**	**Gene/description**	**Mutation/annotation**	**Impact**	**Frequency**
CV725_RS00040	NA	site-specific DNA-methyltransferase, pseudogene	(G)$_{14\rightarrow13}$ (1453/2053 nt)	phase ‘off’ to phase ’on’	91.6%
CV725_RS00720 *CV725_RS00725*	HP1321 HP1322	DS of HP1321 conserved hypothetical ATP-binding protein and US of HP1322 hypothetical protein	(ATACATAA)$_{10\rightarrow5}$ intergenic (-194/+7)	unknown	99.3%
CV725_RS01830		HP1117 family Sel1-like repeat protein	Δ108 bp (231-338/771 nt)	truncation	99.8%
CV725_RS08420	HP1353	Type ISP C-terminal specificity domain, pseudogene	(C)$_{14\rightarrow13}$ (3223/3372 nt)	no effect	92.4%
fur	HP1027	*fur*, ferric uptake regulator	(A)$_{8\rightarrow9}$ (55/453 nt)	Ile18-fs	100%
CV725_RS05120	HP0771	hypothetical protein	Δ27 bp (68-94/738 nt)	in-frame deletion	100%
CV725_RS05665	HP0659	SurA N-terminal domain protein; predicted periplasmic peptidyl-prolyl cis-trans isomerase.	codon-398 (CGC→TGC)	Arg398Cys	100%

bacitracin-resistant isolate of Δ*fapH pflA** strain (H154) – SNPs not present in Δ*fapH pflA** parental strain					
**NCBI designation**	**26695 locus tag**	**Gene/description**	**Mutation/annotation**	**Impact**	**Frequency**
CV725_RS00040	NA	site-specific DNA-methyltransferase, pseudogene	$(G)_{14\rightarrow12}$ (1452/2053 nt)	no effect	94.5%
CV725_RS00720 *CV725_RS00725*	HP1321 HP1322	DS of HP1321 conserved hypothetical ATP-binding protein and US of HP1322 hypothetical protein.	$(ATACATAA)_{10\rightarrow5}$ intergenic (-194/+7)	unknown	99%
CV725_RS08420	HP1353	Type ISP C-terminal specificity domain, pseudogene	$(C)_{14\rightarrow13}$ (3223/3372 nt)	no effect	90.1%
fur	HP1027	*fur*, ferric uptake regulator	C→T ($\underline{G}$AC→$\underline{A}$AC)	Asp135Asn	99.7%
CV725_RS05120	HP0771	hypothetical protein.	Δ27 bp (68-94/738 nt)	truncation	100%
hefC	HP0607	efflux RND transporter permease subunit HefC.	codon-808 (G$\underline{C}$T→G$\underline{T}$T)	Ala808Val	99.7%

bacitracin-resistant isolate of Δ*fapH pflA** strain (H155) – SNPs not present in Δ*fapH pflA** parental strain					
**NCBI designation**	**26695 locus tag**	**Gene/description**	**Mutation/annotation**	**Impact**	**Frequency**
CV725_RS00040	NA	site-specific DNA-methyltransferase, pseudogene	$(G)_{14\rightarrow13}$ (1453/2053 nt)	phase 'off' to phase 'on'	92.2%
CV725_RS00385 *mnmE*	HP1453	outer membrane beta-barrel protein/tRNA uridine-5-carboxymethylaminomethyl (34) synthesis GTPase MnmE	$(T)_{17\rightarrow16}$ intergenic (-105/+113)	unknown	80.5%
CV725_RS00720 *CV725_RS00725*	HP1321 HP1322	DS of HP1321 conserved hypothetical ATP-binding protein and US of HP1322 hypothetical protein.	$(ATACATAA)_{10\rightarrow5}$ intergenic (-194/+7)	unknown	99.5%
CV725_RS08420	HP1353	Type ISP C-terminal specificity domain, pseudogene	$(C)_{14\rightarrow13}$ (3223/3372 nt)	no effect	91.4%
CV725_RS03700	HP0289	vacuolating cytotoxin domain-containing protein.	Codon-670 ($\underline{G}$GG→$\underline{A}$GG)	Gly670Arg	100%
fur	HP1027	*fur*, ferric uptake regulator	$(A)_{8\rightarrow9}$ (55/453 nt)	Ile18-fs	94.9%
CV725_RS05120	HP0771	hypothetical protein.	$(T)_{8\rightarrow9}$ (59/738 nt)	Phe19-fs	94.7%
CV725_RS07860	HP1581	*llm*, methicillin resistance protein	codon-43 ($\underline{C}$CA→$\underline{A}$CA)	Pro43Thr	100%

bacitracin-resistant isolate of Δ*fapH pflA** strain (H156) – SNPs not present in Δ*fapH pflA** parental strain					
**NCBI designation**	**26695 locus tag**	**Gene/description**	**Mutation/annotation**	**Impact**	**Frequency**
CV725_RS00040	NA	site-specific DNA-methyltransferase, pseudogene	$(G)_{14\rightarrow13}$ (1453/2053 nt)	phase 'off' to phase 'on'	90.4%
CV725_RS00720 *CV725_RS00725*	HP1321 HP1322	DS of HP1321 conserved hypothetical ATP-binding protein and US of HP1322 hypothetical protein.	$(ATACATAA)_{10\rightarrow5}$ intergenic (-194/+7)	unknown	99.7%
rpsR *babA*	HP1244 HP1243	DS of HP1244 rpsR 30S ribosomal protein S18 and US of HP1243 outer membrane protein *babA*	$(A)_{13\rightarrow14}$ intergenic (+195/-132)	unknown	80.3%

CV725_RS08420	HP1353	Type ISP C-terminal specificity domain, pseudogene	$(C)_{14\rightarrow13}$ (3223/3372 nt)	no effect	89.8%
fur	HP1027	*fur*, ferric uptake regulator	C→T (GAC→AAC)	Asp135Asn	100%
CV725_RS05120	HP0771	hypothetical protein.	Δ27 bp (68-94/738 nt)	truncation	100%
hefC	HP0607	efflux RND transporter permease subunit HefC.	codon-71 (GGG→AGG)	Gly71Arg	100%
**bacitracin-resistant isolate of Δ*fapH pflA** strain (H157) – SNPs not present in Δ*fapH pflA** parental strain**					
**NCBI designation**	**26695 locus tag**	**Gene/description**	**Mutation/annotation**	**Impact**	**Frequency**
CV725_RS00040	NA	site-specific DNA-methyltransferase, pseudogene	$(G)_{14\rightarrow13}$ (1453/2053 nt)	phase 'off' to phase 'on'	93.5%
CV725_RS00720 *CV725_RS00725*	HP1321 HP1322	DS of HP1321 conserved hypothetical ATP-binding protein and US of HP1322 hypothetical protein.	$(ATACATAA)_{10\rightarrow5}$ intergenic (-194/+7)	unknown	98.8%
CV725_RS08420	HP1353	Type ISP C-terminal specificity domain, pseudogene	$(C)_{14\rightarrow13}$ (3223/3372 nt)	no effect	88.1%
fur	HP1027	*fur*, ferric uptake regulator	$(A)_{8\rightarrow9}$ (55/453 nt)	Ile18-fs	94.8%
CV725_RS05120	HP0771	hypothetical protein.	$(T)_{8\rightarrow9}$ (59/738 nt)	Phe19-fs	93.3%
CV725_RS06910	HP0947	hypothetical protein	+T coding (173/363 nt)	Lys58-fs	100%
**bacitracin-resistant isolate of Δ*fapH pflA** strain (H158) – SNPs not present in Δ*fapH pflA** parental strain**					
**NCBI designation**	**26695 locus tag**	**Gene/description**	**Mutation/annotation**	**Impact**	**Frequency**
CV725_RS00040	NA	site-specific DNA-methyltransferase, pseudogene	$(G)_{14\rightarrow13}$ (1453/2053 nt)	phase 'off' to phase 'on'	96.5%
CV725_RS00720 *CV725_RS00725*	HP1321 HP1322	DS of HP1321 conserved hypothetical ATP-binding protein and US of HP1322 hypothetical protein.	$(ATACATAA)_{10\rightarrow5}$ intergenic (-194/+7)	unknown	100%
CV725_RS02330 *CV725_RS02335*	HP1370 NA	biotin synthase/type III restriction enzyme.	$(C)_{13\rightarrow14}$ intergenic (-243/-51)	unknown	85%
fur	HP1027	*fur*, ferric uptake regulator	$(A)_{8\rightarrow9}$ (55/453 nt)	Ile18-fs	99.5%
CV725_RS05120	HP0771	hypothetical protein.	Δ27 bp (68-94/738 nt)	in-frame deletion	100%
hefC	HP0607	efflux RND transporter permease subunit HefC.	codon-642 (AAT→GAT)	Asn64Asp	100%
**bacitracin-resistant isolate of Δ*fapH pflA** strain (H159) – SNPs not present in Δ*fapH pflA** parental strain**					
**NCBI designation**	**26695 locus tag**	**Gene/description**	**Mutation/annotation**	**Impact**	**Frequency**
CV725_RS00040	NA	site-specific DNA-methyltransferase, pseudogene	$(G)_{14\rightarrow13}$ (1453/2053 nt)	phase 'off' to phase 'on'	95.9%
CV725_RS00720 *CV725_RS00725*	HP1321 HP1322	DS of HP1321 conserved hypothetical ATP-binding protein and US of HP1322 hypothetical protein.	$(ATACATAA)_{10\rightarrow5}$ intergenic (-194/+7)	unknown	99.1%

CV725_RS02330 *CV725_RS02335*	HP1370 NA	biotin synthase/type III restriction enzyme.	$(C)_{13\rightarrow14}$ intergenic (-243/-51)	unknown	88.3%
CV725_RS08420	HP1353	Type ISP C-terminal specificity domain, pseudogene	$(C)_{14\rightarrow13}$ (3223/3372 nt)	no effect	89.4%
fur	HP1027	*fur*, ferric uptake regulator	$(A)_{8\rightarrow9}$ (55/453 nt)	Ile18-fs	98.1%
CV725_RS05120	HP0771	hypothetical protein.	$(T)_{8\rightarrow9}$ (59/738 nt)	Phe19-fs	94.8%
**bacitracin-resistant isolate of Δ*fapH pflA** strain (H160) – SNPs not present in Δ*fapH pflA** parental strain**					
**NCBI designation**	**26695 locus tag**	**Gene/description**	**Mutation/annotation**	**Impact**	**Frequency**
CV725_RS00040	NA	site-specific DNA-methyltransferase, pseudogene	$(G)_{14\rightarrow13}$ (1453/2053 nt)	phase 'off' to phase 'on'	94.2%
CV725_RS00720 *CV725_RS00725*	HP1321 HP1322	DS of HP1321 conserved hypothetical ATP-binding protein and US of HP1322 hypothetical protein.	$(ATACATAA)_{10\rightarrow5}$ intergenic (-194/+7)	unknown	99%
CV725_RS02330 *CV725_RS02335*	HP1370 NA	biotin synthase/type III restriction enzyme.	$(C)_{13\rightarrow14}$ intergenic (-243/-51)	unknown	81.7%
CV725_RS08420	HP1353	Type ISP C-terminal specificity domain, pseudogene	$(C)_{14\rightarrow11}$ pseudogene (3222-3224/ 3372 nt)	no effect	81.9%
fur	HP1027	*fur*, ferric uptake regulator	C→T (GAC→AAC)	Asp135Asn	100%
CV725_RS05120	HP0771	hypothetical protein	Δ27 bp coding (68-94/738 nt)	truncation	100%
CV725_RS06720 *alpA*	HP0911 HP0912	DS of ATP-dependent helicase/US of Hop family adhesin AlpA (HopC).	$(T)_{14\rightarrow15}$ intergenic (+380/-206)	unknown	85%
CV725_RS06910	HP0947	hypothetical protein (No hits for HP designation)	codon-114 (GCC→ACC)	Ala114Thr	100%
**bacitracin-resistant isolate of Δ*fapH pflA** strain (H161) – SNPs not present in Δ*fapH pflA** parental strain**					
**NCBI designation**	**26695 locus tag**	**Gene/ description**	**Mutation/annotation**	**Impact**	**Frequency**
CV725_RS00040	NA	site-specific DNA-methyltransferase, pseudogene	$(G)_{14\rightarrow13}$ (1453/2053 nt)	phase 'off' to phase 'on'	85.9%
CV725_RS00720 *CV725_RS00725*	HP1321 HP1322	DS of HP1321 conserved hypothetical ATP-binding protein and US of HP1322 hypothetical protein.	$(ATACATAA)_{10\rightarrow5}$ intergenic (-194/+7)	unknown	99.7%
CV725_RS00915	HP1284	glycosyltransferase family 9 protein ADP-heptose:LPS heptosyltransferase	codon-236 (GGC→GTC)	Gly236Val	98.9%
CV725_RS02330 *CV725_RS02335*	HP1370 NA	biotin synthase/type III restriction enzyme.	$(C)_{13\rightarrow14}$ intergenic (-243/-51)	unknown	84.6%
CV725_RS08420	HP1353	Type ISP C-terminal specificity domain, pseudogene	$(C)_{14\rightarrow13}$ (3223/3372 nt)	no effect	91.8%
fur	HP1027	*fur*, ferric uptake regulator	$(A)_{8\rightarrow9}$ (55/453 nt)	Ile18-fs	96.8%
CV725_RS05120	HP0771	hypothetical protein	$(T)_{8\rightarrow9}$ (59/738 nt)	Phe19-fs	93.1%
***H. pylori* B128 Δ*fur*-7 – SNPs not present in wild-type *H. pylori* B128**					
**NCBI designation**	**26695 locus tag**	**Gene/ description**	**Mutation/annotation**	**Impact**	**Frequency**

CV725_RS00720<br>*CV725_RS00725*	HP1321<br>HP1322	DS of HP1321 conserved hypothetical ATP-binding protein and US of HP1322 hypothetical protein.	(ATACATAA)$_{10\rightarrow5}$<br>intergenic (-194/+7)	unknown	100%
CV725_RS02305<br>*CV725_RS02315*	HP0427<br>23S rRNA	US of *hp0427* (hypothetical protein), US of 23S rRNA gene	A→G<br>intergenic (-235/-735)	unknown	89.7%
fur	HP1027	*fur*, ferric uptake regulator	Δ436 bp	gene deletion	100%
CV725_RS04600<br>*CV725_RS04605*	HP0875<br>HP0876	US of *katA,* US of *frpB*	(A)$_{5\rightarrow6}$<br>intergenic (-34/-290)	unknown	93.9%
motA	HP0815	*motA*	codon-201<br>(GGC→GAC)	Gly201Asp	99.3%
CV725_RS06350	HP0527	*cag* pathogenicity island protein (*cag7*)	codon-1226<br>(TTA→CTA)<br>codon-752<br>(ACC→ACG)<br>codon-742<br>(GCT→GCC)	Leu1226Leu<br>Thr752Thr<br>Ala742Ala	99.5%<br>89.5%<br>80.8%
CV725_RS06500	HP0499	*pldA*, phospholipase A pseudogene	(G)$_{10\rightarrow8}$<br>(683/1070 nt)	phase 'off' to phase 'on'	98.2%
CV725_RS07515<br>*queF*	HP1412<br>HP1413	DS of HP1412 hypothetical protein and DS of *queF*	(C)$_{11\rightarrow10}$<br>intergenic (+11/+121)	unknown	96.1%
H. pylori **B128 Δ*fur*-10 – SNPs not present in wild-type *H. pylori* B128**					
**NCBI designation**	**26695 locus tag**	**Gene/ description**	**Mutation/annotation**	**Impact**	**Frequency**
CV725_RS00720<br>*CV725_RS00725*	HP1321<br>HP1322	DS of HP1321 conserved hypothetical ATP-binding protein and US of HP1322 hypothetical protein.	(ATACATAA)$_{10\rightarrow5}$<br>intergenic (-194/+7)	unknown	99.6%
rpsS<br>*rplV*		30S ribosomal protein S19/50S ribosomal protein L22	(G)5→4<br>intergenic (+5/-5)	unknown	99.6%
CV725_RS00895	HP1288	pantothenate kinase	-T (81/351 nt)	Phe27-fs	99.3%
fur	HP1027	*fur*, ferric uptake regulator	Δ436 bp	gene deletion	100%
CV725_RS04600<br>*CV725_RS04605*	HP0875<br>HP0876	US of *katA,* US of *frpB*	(A)$_{5\rightarrow6}$<br>intergenic (-34/-290)	unknown	91.9%
motA	HP0815	*motA*	codon-201<br>(GGC→GAC)	Gly201Asp	100%
CV725_RS06350	HP0527	*cag* pathogenicity island protein (*cag7*)	codon-1226<br>(TTA→CTA)<br>codon-936<br>(ACG→ACC)<br>codon-752<br>(ACC→ACG)<br>codon-742<br>(GCT→GCC)	Leu1226Leu<br>Thr936Thr<br>Thr752Thr<br>Ala742Ala	99.6%<br>97.1%<br>95.4%<br>96.3%
CV725_RS06500	HP0499	*pldA*, phospholipase A pseudogene	(G)$_{10\rightarrow7}$<br>(683/1070 nt)	remains phase 'off'	85.1%
CV725_RS07515<br>*queF*	HP1412<br>HP1413	DS of HP1412 hypothetical protein and DS of *queF*	(C)$_{11\rightarrow10}$<br>intergenic (+11/+121)	unknown	96.1%

***H. pylori* B128 Δ*fapH pflA** Δ*fur*-5 – SNPs not present in Δ*fapH pflA** parental strain**					
**NCBI designation**	**26695 locus tag**	**Gene/ description**	**Mutation/annotation**	**Impact**	**Frequency**
CV725_RS00040	NA	site-specific DNA-methyltransferase, pseudogene	$(G)_{14\rightarrow13}$ (1453/2053 nt)	phase 'off' to phase 'on'	89.5%
CV725_RS00720<br>*CV725_RS00725*	HP1321<br>HP1322	DS of HP1321 conserved hypothetical ATP-binding protein and US of HP1322 hypothetical protein.	$(ATACATAA)_{10\rightarrow5}$<br>intergenic (-194/+7)	unknown	99.5%
maf<br>*CV725_RS01150*	HP1239<br>HP1240	DS of *maf*, US of *hp1239* (hypothetical protein)	$(A)_{13\rightarrow16}$<br>intergenic (+74/+381)	unknown	83.9%
CV725_RS02330<br>*CV725_RS02335*	HP1370<br>NA	biotin synthase/type III restriction enzyme.	$(C)_{13\rightarrow14}$<br>intergenic (-243/-51)	unknown	88.4%
CV725_RS08420	HP1353	Type ISP C-terminal specificity domain, pseudogene	$(C)_{14\rightarrow13}$<br>(3223/3372 nt)	no effect	89.3%
CV725_RS03080	HP0164	*arsS*, histidine kinase	$(C)_{11\rightarrow12}$<br>coding (1261/1281 nt)	Pro424-fs	86.7%
fur	HP1027	*fur*, ferric uptake regulator	Δ436 bp	gene deletion	100%
CV725_RS04785	HP0838	*fapH*, flagellar-associate protein H	Δ490 bp	gene deletion	100%
CV725_RS05175<br>*rny*	HP0761	5-formyltetrahydrofolate cyclo-ligase ribonuclease Y	codon-208 (TAC→TAT)	Tyr208Tyr	99.2%
CV725_RS07655<br>*labA*	HP0026<br>HP0025	citrate synthase/Hop family adhesin LabA	(T)17→16<br>intergenic (+379/-149)	unknown	83.3%
***H. pylori* B128 Δ*fapH pflA** Δ*fur*-9 – SNPs not present in Δ*fapH pflA** parental strain**					
CV725_RS00040	NA	site-specific DNA-methyltransferase, pseudogene	$(G)_{14\rightarrow13}$ (1453/2053 nt)	phase 'off' to phase 'on'	89.7%
CV725_RS00720<br>*CV725_RS00725*	HP1321<br>HP1322	DS of HP1321 conserved hypothetical ATP-binding protein and US of HP1322 hypothetical protein.	$(ATACATAA)_{10\rightarrow5}$<br>intergenic (-194/+7)	unknown	99.6%
CV725_RS08420	HP1353	Type ISP C-terminal specificity domain, pseudogene	$(C)_{14\rightarrow13}$<br>(3223/3372 nt)	no effect	89.4%
CV725_RS03080	HP0164	*arsS*, histidine kinase	$(C)_{11\rightarrow12}$<br>coding (1261/1281 nt)	Pro424-fs	80.3%
fur	HP1027	*fur*, ferric uptake regulator	Δ436 bp	gene deletion	100%
CV725_RS04785	HP0838	*fapH*, flagellar-associated protein H	Δ490 bp	gene deletion	100%
CV725_RS05175<br>*rny*	HP0761	5-formyltetrahydrofolate cyclo-ligase ribonuclease Y	codon-208 (TAC→TAT)	Tyr208Tyr	100%
CV725_RS05825	HP0628	sel1 repeat family protein	(C)7→6<br>coding (505/1059 nt)	Gly169-fs	95.6%
CV725_RS06350	HP0527	cag pathogenicity island protein (Cag7)	codon-936<br>(ACG→ACC)	Thr936Thr	96.9%
CV725_RS07655<br>*labA*	HP0026<br>HP0025	citrate synthase/Hop family adhesin LabA	(T)17→16<br>intergenic (+379/-149)	unknown	83.1%

**Δ*fapH*-2 – SNPs not present in wild-type *H. pylori* B128**					
CV725_RS00720<br>*CV725_RS00725*	HP1322<br>HP1321	DS of HP1321 conserved hypothetical ATP-binding protein and US of HP1322 hypothetical protein.	(ATACATAA)$_{10\rightarrow5}$<br>intergenic (-194/+7)	unknown	99.0%
CV725_RS02305<br>*CV725_RS02315*	HP0427<br>23S rRNA	US of *hp0427* (hypothetical protein), US of 23S rRNA gene	C→T<br>intergenic (-235/-735)	unknown	81.6%
CV725_RS02465	HP1375	N-6 DNA methylase	(C)$_{14\rightarrow13}$<br>pseudogene (3224/3372 nt)	restore full-length ORF	91.8%
CV725_RS03080	HP0164	*arsS*, histidine kinase	(C)$_{11\rightarrow12}$<br>coding (1261/1281 nt)	Pro424-fs	85.7%
CV725_RS04600<br>*CV725_RS04605*	HP0875<br>HP0876	US of HP0875 *katA,* US of HP0876 *frpB*	(A)$_{5\rightarrow6}$<br>intergenic (-34/-290)	unknown	93.0%
CV725_RS04785	HP0838	*fapH*, flagellar-associated protein H	Δ490 bp	gene deletion	100%
CV725_RS06500	HP0499	*pldA*, phospholipase A pseudogene	(G)$_{10\rightarrow9}$<br>(683/1070 nt)	remains phase ’off’	94.8%
CV725_RS07515<br>*queF*	HP1412<br>HP1413	DS of HP1412 hypothetical protein and DS of queF	(C)$_{11\rightarrow10}$<br>intergenic (+11/+121)	unknown	96.4%
lpxF	HP1580	membrane-associated phospholipid phosphatase	G→A<br>(TGG→TGA)	W14stop	100%
**Δ*fapH*-11 – SNPs not present in wild-type *H. pylori* B128**					
serS	HP1480	serine-tRNA ligase	C→G<br>(TCC→TCG)	Ser352Ser	99.6%
CV725_RS00720<br>*CV725_RS00725*	HP1322<br>HP1321	DS of HP1321 conserved hypothetical ATP-binding protein and US of HP1322 hypothetical protein.	(ATACATAA)$_{10\rightarrow5}$<br>intergenic (-194/+7)	unknown	98.2%
CV725_RS02305<br>*CV725_RS02315*	HP0427<br>23S rRNA	US of *hp0427* (hypothetical protein), US of 23S rRNA gene	+32 bp<br>intergenic (-234/-736)	unknown	92.3%
CV725_RS02465	HP1375	N-6 DNA methylase	(C)$_{14\rightarrow13}$<br>pseudogene (3224/3372 nt)	restore full-length ORF	90.5%
CV725_RS04600<br>*CV725_RS04605*	HP0875<br>HP0876	US of HP0875 *katA,* US of HP0876 *frpB*	(A)$_{5\rightarrow6}$<br>intergenic (-34/-290)	unknown	95.6%
CV725_RS04785	HP0838	*fapH*, flagellar-associated protein H	Δ490 bp	gene deletion	100%
CV725_RS06350	HP0527	cag pathogenicity island protein (Cag7)	codon-752<br>(ACC→ACG)<br>codon-742<br>(GCT→GCC)	Thr752Thr<br><br>Ala742Ala	91.2%<br><br>95.5%
CV725_RS06500	HP0499	*pldA*, phospholipase A pseudogene	(G)$_{10\rightarrow9}$<br>(683/1070 nt)	remains phase ’off’	95.1%
CV725_RS07515<br>*queF*	HP1412<br>HP1413	DS of HP1412 hypothetical protein and DS of queF	(C)$_{11\rightarrow10}$<br>intergenic (+11/+121)	unknown	94.5%
lpxF	HP1580	membrane-associated phospholipid phosphatase	G→A<br>(TGG→TGA)	W14stop	99.3%
**Δ*fapH*-4 – SNPs not present in wild-type *H. pylori* B128**					

CV725_RS00720 *CV725_RS00725*	HP1322 HP1321	DS of HP1321 conserved hypothetical ATP-binding protein and US of HP1322 hypothetical protein.	$(ATACATAA)_{10\rightarrow5}$ intergenic (-194/+7)	unknown	99.6%
CV725_RS02305 *CV725_RS02315*	HP0427 23S rRNA	US of *hp0427* (hypothetical protein), US of 23S rRNA gene	A→G intergenic (-235/-735)	unknown	86.2%
CV725_RS02465	HP1375	N-6 DNA methylase	$(C)_{14\rightarrow13}$ pseudogene (3224/3372 nt)	restore full-length ORF	92.3%
CV725_RS03080	HP0164	*arsS*, histidine kinase	$(C)_{11\rightarrow12}$ coding (1261/1281 nt)	Pro424-fs	80.8%
CV725_RS04600 *CV725_RS04605*	HP0875 HP0876	US of HP0875 *katA,* US of HP0876 *frpB*	$(A)_{5\rightarrow6}$ intergenic (-34/-290)	unknown	92.8%
CV725_RS04785	HP0838	*fapH*, flagellar-associated protein H	Δ490 bp	gene deletion	100%
CV725_RS06350	HP0527	cag pathogenicity island protein (cag7)	codon-742 (GC<u>T</u>→GC<u>C</u>)	Ala742Ala	82.3%
CV725_RS06500	HP0499	*pldA*, phospholipase A pseudogene	$(G)_{10\rightarrow9}$ (683/1070 nt)	remains phase 'off'	93.3%
CV725_RS07515 *queF*	HP1412 HP1413	DS of HP1412 hypothetical protein and DS of queF	$(C)_{11\rightarrow10}$ intergenic (+11/+121)	unknown	98.0%
**Δ*fapH*-9 – SNPs not present in wild-type *H. pylori* B128**					
CV725_RS00720 *CV725_RS00725*	HP1322 HP1321	DS of HP1321 conserved hypothetical ATP-binding protein and US of HP1322 hypothetical protein.	$(ATACATAA)_{10\rightarrow5}$ intergenic (-194/+7)	unknown	100%
CV725_RS02305 *CV725_RS02315*	HP0427 23S rRNA	US of *hp0427* (hypothetical protein), US of 23S rRNA gene	+32 bp intergenic (-234/-736)	unknown	89.3%
CV725_RS02465	HP1375	N-6 DNA methylase	$(C)_{14\rightarrow13}$ pseudogene (3224/3372 nt)	restore full-length ORF	84.3%
CV725_RS04600 *CV725_RS04605*	HP0875 HP0876	US of HP0875 *katA,* US of HP0876 *frpB*	$(A)_{5\rightarrow6}$ intergenic (-34/-290)	unknown	97.1%
CV725_RS04780	HP0839	outer membrane protein P1 (ompP1)	altered 30 bp sequence coding (494-523/1764 nt	Asn-165 through Thr-175 altered	100%
CV725_RS04785	HP0838	*fapH*, flagellar-associated protein H	Δ490 bp	gene deletion	100%
CV725_RS06350	HP0527	cag pathogenicity island protein (Cag7)	codon-752 (AC<u>C</u>→AC<u>G</u>)	Thr752Thr	82.6%
CV725_RS06500	HP0499	*pldA*, phospholipase A pseudogene	$(G)_{10\rightarrow9}$ (683/1070 nt)	remains phase 'off'	95.2%
CV725_RS07515 *queF*	HP1412 HP1413	DS of HP1412 hypothetical protein and DS of queF	$(C)_{11\rightarrow10}$ intergenic (+11/+121)	unknown	98%
CV725_RS07950	HP1562	iron(III) ABC transporter, periplasmic iron-binding protein (ceuE)	codon-180 (GA<u>G</u>→GA<u>T</u>)	Glu180Asp	100%

[a]"fs" indicates frameshift

[b]Frequency is the number of times the SNP was identified relative to the total number of reads for that sequence. Only SNPs that had a frequency ≥80% are shown.

[c]'DS' and 'US' are abbreviations for sequences that are 'downstream' and 'upstream' of the gene, respectively.
